# The gut resistome in poultry production: microbial ecology, antibiotic use, and sustainable control approaches

**DOI:** 10.3389/fmicb.2026.1768747

**Published:** 2026-02-24

**Authors:** Zonghui Jian, Yuwei Qian, Shichun He, Ruohan Zhao, Ke Li, Jinlong Cha, Zhijun Ning, Yanlin Ye, Zhipeng Bao, Kun Wang, Changrong Ge, Junjing Jia, Tengfei Dou, Yuanyuan Hu, Xiaoming He, Xiannian Zi

**Affiliations:** 1 Yunnan Vocational and Technical College of Agriculture, Kunming, China; 2 Yunnan Agricultural University, Kunming, China; 3 Institute of Poultry Science, Yunnan Academy of Animal Husbandry and Veterinary Sciences, Kunming, Yunnan Province, China

**Keywords:** antibiotic resistance genes, gut microbiome, mobile genetic elements, poultry gut resistome, resistome–microbiome–metabolome axis

## Abstract

Antibiotics remain central to modern poultry production, but their long-term and sometimes poorly managed use has markedly altered gut microbial ecology, effectively transforming the intestine into a substantial reservoir of antibiotic resistance genes (ARGs). In poultry, the composition of ARGs reflects not only resistant bacterial taxa but also the activity of mobile genetic elements, shifts in gut metabolic conditions, and features of the surrounding production system. This review synthesizes current understanding of both the structural and functional features of the poultry resistome, with particular attention to key bacterial hosts and the mobile genetic elements they carry. We further evaluate how different antibiotic-use patterns and additional co-selective pressures alter microbial communities and contribute to the persistence of ARGs. We also delineate the major transmission pathways that link breeder flocks, hatcheries, production facilities, and manure management, and interpret these connections within a One Health perspective. Particular emphasis is placed on microbial and nutritional interventions that influence gut microbial interactions, epithelial barrier integrity, and metabolic signaling. Drawing on these findings, we propose a resistome–microbiome–metabolome axis that links microbial taxa, resistance elements, and key metabolic signals, offering a conceptual framework for developing more targeted antimicrobial resistance mitigation strategies in poultry systems.

## 1 Introduction

Since antibiotics were first introduced into livestock production in the 1940s, they have come to play a central role in disease control and productivity improvement. Yet their prolonged, repeated, and sometimes poorly regulated use has driven the emergence and spread of antibiotic-resistant bacteria (ARB) and resistance genes (ARGs), contributing to the global antimicrobial resistance crisis (McEwen and Collignon, 2018; Van Boeckel et al., 2019). Poultry production accounts for a substantial share of veterinary antibiotic use, as antimicrobials are routinely administered for treatment, disease prevention, and growth promotion (de Mesquita Souza Saraiva et al., 2022; Government Accountability Office, 2017). More than 70% of commercial poultry encounter antibiotics at some stage of production, and an estimated 60–90% of these drugs are excreted either unmetabolized or as active metabolites (Landers et al., 2012; McEwen and Collignon, 2018). Such residues alter gut microbial composition, promote the enrichment of ARGs, and facilitate horizontal gene transfer (HGT) among gut microbes (Jian et al., 2021; Wang et al., 2017). As a result, the poultry gut now functions as a concentrated ecological reservoir of ARGs. These genes can disperse into soil, water, and air via fecal waste, posing risks not only to animals but also to environmental and public health (Berendonk et al., 2015; Phan et al., 2024).

The concept of the “resistome”, first proposed by Wright, refers to the full range of known and potential ARGs together with the regulatory and mobilization elements that govern their expression and movement, such as chromosomal genes, plasmids, integrons, transposons, and other mobile genetic elements (MGEs; Wright, 2007). Initial antimicrobial resistance (AMR) studies emphasized phenotypic resistance, but advances in metagenomics, metatranscriptomics, and metabolomics have shifted attention toward ecological, network-based interpretations. This systems perspective underscores how microbial taxa, ARGs, MGEs, and environmental factors interact in complex ways to shape resistance dynamics. In poultry systems, the gut resistome reflects the combined influence of host physiology, microbial community composition, antibiotic pressure, co-selective factors, and the metabolic state of the gut (Feng et al., 2021). Factors such as diet, antibiotic-use patterns, housing conditions, and microbial interactions further shape ARGs diversity, abundance, and mobility.

The poultry gut resistome is largely shaped by resistance determinants targeting commonly used veterinary antimicrobials such as tetracyclines, aminoglycosides, macrolides, and β-lactams (Redman-White et al., 2023). These genes are frequently carried by both opportunistic pathogens and commensal species, such as *Escherichia coli, Enterococcus, Bacteroides*, and *Clostridium*, many of which harbor various MGEs that facilitate frequent horizontal transfer (Stokes and Gillings, 2011). Antibiotic exposure may also trigger stress responses, SOS signaling, and quorum-sensing pathways, which can elevate ARGs expression and enhance their transfer potential (Forslund et al., 2013). ARGs dissemination in poultry involves multiple hosts and environments, linking animals, production facilities, and surrounding ecosystems within an interconnected One Health transmission network. The close correspondence between environmental resistomes and gut-derived ARGs profiles suggests substantial gene exchange and co-evolution across these compartments (Qiao et al., 2018). At the same time, microbial and nutritional strategies, such as probiotics, plant-derived bioactive compounds, short chain fatty acids (SCFAs), and ecological feed additives, can modulate the gut community structure, strengthen mucosal barriers, and attenuate MGE-mediated transfer, making them potential tools for mitigating the resistome (Etienne et al., 2025; Gadde et al., 2017; Liang et al., 2025).

**Figure 1:**
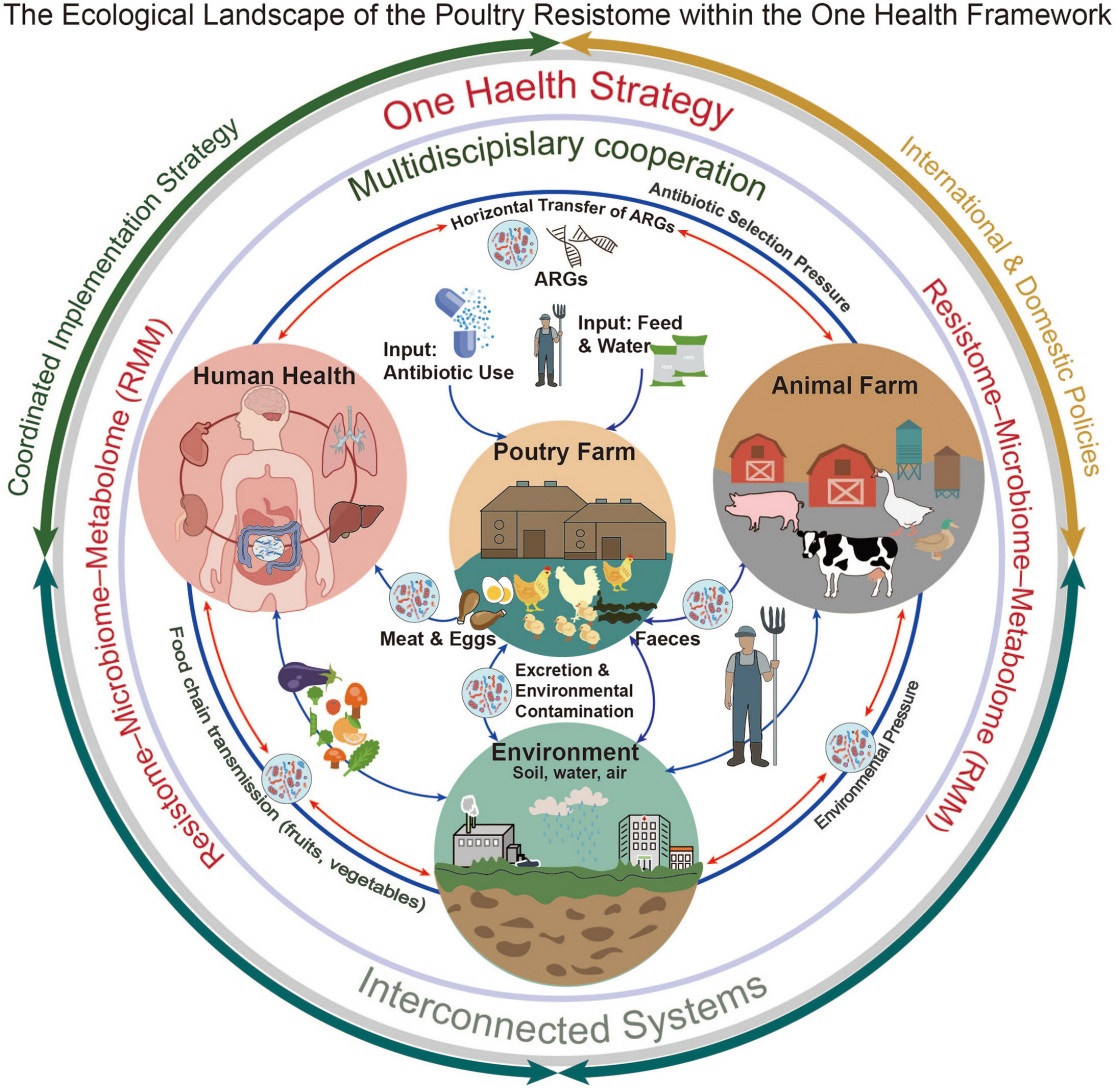
This schematic illustrates key ecological processes shaping the poultry resistome across animal, environmental, and human domains. Demonstrated pathways include the introduction of antibiotics and pre-existing ARGs into the poultry gut via feed, water, and antimicrobial use, as well as the role of environmental compartments as reservoirs for ARGs persistence. Associative pathways, supported primarily by correlative field and surveillance studies, describe the links between environmental matrices (e.g., litter, water, and dust) and recontamination of feed and water, contributing to ongoing resistome circulation. The spillback loop, depicting bidirectional exchange of ARGs among poultry, environment, and humans, is presented as a hypothetical or inference-based pathway that integrates observed associations rather than direct causal evidence. Overall, the figure provides an evidence-informed conceptual overview of interconnected health pathways potentially contributing to ARGs amplification and dissemination within a One Health framework.

In this review, we introduce the resistome–microbiome–metabolome (RMM) axis, a framework that moves beyond conventional resistome profiling by highlighting metabolic and ecological forces that drive ARGs amplification. RMM axis is an evidence-informed conceptual framework rather than a universally validated mechanistic model. Associations between microbial community composition and ARGs abundance are supported by multiple poultry *in vivo* studies, whereas links involving microbial metabolites, ARGs expression, and HGT are primarily derived from poultry *in vitro* experiments, non-poultry animal models, or ecological inference. Most traditional studies relate ARGs to host taxa or MGEs, but less attention has been given to how microbial metabolites, such as SCFAs, lactate, or bile-acid derivatives, affect gene transfer, stress responses, or ecological competition (Shilov et al., 2025; Yang et al., 2022). By combining metabolomic and transcriptomic data, the RMM axis helps identify potential regulatory nodes, including: (i) metabolite-mediated modulation of MGEs activity (e.g., butyrate-dependent repression of conjugation), (ii) metabolic shifts that alter ecological niches and ARGs–host fitness, and (iii) co-localization of metabolic and resistance genes that form functional resistance modules (Kumar et al., 2025; Wang et al., 2020; Wu et al., 2022). By integrating host phylogeny, MGEs mobility, metabolic outputs, and ecological feedbacks, the RMM axis offers a mechanistic basis for predicting ARGs-related risks and designing targeted nutritional or microecological interventions (Figure 1). Unlike many earlier reviews centered mainly on microbial taxonomy or ARGs abundance, our analysis emphasizes the functional co-maintenance of MGEs and metabolic pathways, considers SCFAs-mediated modulation of HGT, and situates resistome dynamics within a One Health environmental feedback framework. This integrated perspective helps inform the development of more precise nutritional and microecological strategies aimed at limiting ARGs dissemination in poultry systems.

## 2 Composition and functional features of the poultry gut resistome

### 2.1 Taxonomic driving factors

Several core host taxa dominate the amplification and maintenance of ARGs within the poultry gut. Evidence from both metagenomic datasets and culture-based work consistently identifies *Escherichia* (especially *E. coli*), *Enterococcus, Bacteroides*, and certain *Lactobacillus* groups as major ARGs carriers (Yang et al., 2022). While these genera contribute important fermentative and nutritional functions, their genomic flexibility and strong association with MGEs enable them to act as efficient reservoirs and disseminators of ARGs when selective pressures are present (Meng et al., 2025). Large metagenomic datasets indicate that *E. coli* genomes frequently contain combinations of *bla, tet*, and *sul* genes, creating stable and highly interconnected “ARGs hubs” in the gut microbial network (Xu Y. et al., 2022). The anatomical separation of different gut regions creates distinct ecological conditions, which in turn shape resistome structure. In the small intestine, where digesta moves quickly, pH is low, and oxygen levels are limited, fast-growing taxa such as *Lactobacillus* dominate, and ARGs abundance remains relatively low, with many encoding efflux or modification functions (Yue et al., 2024). By contrast, the cecum functions as a strictly anaerobic fermentation chamber, showing slower passage rates, high microbial diversity, and substantial SCFAs production. These features result in 1.5- to 3-fold higher ARGs richness and increased prevalence of plasmid- and integron-associated MGEs, making the cecum the major reservoir and exchange hotspot for mobile resistance elements (Shen et al., 2024; Xu Y. et al., 2022). Broad metagenomic surveys repeatedly show that the cecum contains both higher ARGs richness and abundance compared with the small intestine or feces. High-risk ARGs (*bla* clusters) are often found on specific plasmids or chromosomal regions, underscoring the genomic contexts that support their persistence and mobility. Comparative analyses further show that management practices, especially chronic, low-dose antibiotic exposure in intensive operations can markedly shift cecal microbiota and resistome patterns, leading to the greatest ARGs burden in high-density systems (Pan et al., 2024).

Core host bacteria maintain the resistome through several complementary mechanisms. First, they act as major reservoirs for plasmids and integrons and promote HGT by conjugation, vesicle-mediated transfer, and transduction. Their metabolic activities, such as producing organic acids and SCFAs, modify the gut microenvironment, influencing microbial competition and ARGs expression. Together, these processes form an “eco-genetic network” in which host identity, MGEs mobility, and metabolite dynamics interact to shape how resistance spreads. Thus, beyond measuring ARGs abundance alone, integrating host–gene associations, MGEs contexts, and multi-omics evidence is crucial for meaningful risk assessment (Baker et al., 2023; Yuan et al., 2024).

### 2.2 Functional diversity of the resistome

The poultry gut resistome exhibits broad functional diversity, encompassing resistance to multiple antibiotic classes (Table 1). These genes participate in complex networks linking AMR with microbial metabolism, stress regulation, and MGEs activity. Large metagenomic assemblies and genome reconstructions indicate that ARGs are frequently embedded within gene islands or mobile elements located next to metabolic or stress-related genes (Shilov et al., 2025). For instance, many chicken-derived genomes place ARGs alongside genes involved in carbon and energy metabolism or SCFAs production. This arrangement suggests that resistance traits and metabolic functions may be co-maintained or co-transferred under certain ecological pressures, forming “functional resistance modules”. Evidence from large resistome catalogs derived from hundreds of chicken gut samples supports this idea, showing that many ARGs-containing contigs also include plasmid markers and metabolic genes. These findings suggest that mobile resistance islands commonly encode both AMR determinants and metabolic functions that may shape host–microbe interactions and microbial fitness (Yang et al., 2022).

**Table 1:** Classification and characteristics of ARGs.

**ARGs class**	**Representative genes**	**Primary mechanisms of action**	**Common host bacterial genera**	**Distribution and ecological characteristics in poultry gut**
β-lactams	*blaTEM, blaCTX-M, blaSHV, blaOXA*	Produce β-lactamases that hydrolyze β-lactam antibiotics.	*Escherichia, Enterococcus, Klebsiella*	Detected in both the cecum and small intestine; abundance markedly increases under β-lactam exposure due to selection pressure (Faridah et al., 2023; Yang et al., 2022).
Tetracyclines	*tetA, tetM, tetO, tetQ*	Efflux pump activity and ribosomal protection protein mechanisms.	*Lactobacillus, Bacteroides, Enterococcus*	Highly enriched in the cecum; frequently co-localized with MGEs, indicating strong potential for horizontal transfer (Bai et al., 2024; Juricova et al., 2021).
Macrolides	*ermB, mefA, mphA*	Methylation of the 23S rRNA target site or efflux-mediated resistance.	*Enterococcus, Clostridium, Streptococcus*	Abundance increases with age and exposure to feed-derived macrolides; shows strong association with production-stage antibiotic use (Huang et al., 2024).
Aminoglycosides	*aadA, aph(3′), ant(6′)*	Antibiotic modification via phosphorylation, adenylation, or acetylation.	*E. coli, Enterobacter, Acinetobacter*	Frequently enriched in upper intestinal sections; carried predominantly on plasmids, enabling rapid dissemination (Koorakula et al., 2022).
Sulfonamides	*sul1, sul2, sul3*	Mutations or substitutions in dihydropteroate synthase.	*Escherichia, Bacteroides*	Often co-occurs with class I integrons (*intI1*), suggesting strong horizontal gene transfer potential (Kador et al., 2025).
Quinolones	*qnrA, qnrB, aac(6′)-Ib-cr*	Target protection proteins or modifying enzymes.	*Salmonella, Escherichia, Enterobacter*	High detection rates throughout slaughter and manure-processing chains, posing downstream environmental risks (Rawat et al., 2024).
Integrons and Efflux systems	*acrB, mdfA, intI1, ISCR1*	Efflux pump activity and integron-mediated gene capture and dissemination.	*E. coli, Enterococcus, Bacteroides*	Serve as core nodes in ARGs horizontal transfer; abundance co-varies with residual antibiotics and heavy metals in the environment (Błażejewska et al., 2022).

MGEs provide the molecular machinery that may contribute to rapid ARGs dissemination in the poultry gut (Table 2). Plasmid-centered studies report that poultry isolates of *E. coli, Salmonella*, and *Enterococcus* frequently harbor *Inc*-type conjugative plasmids. These plasmids often carry clinically important ARGs and frequently include complete *tra* transfer systems that promote resistance transfer within and between bacterial genera. For instance, multidrug-resistant *E. coli* from poultry manure in Poland contained *IncC* and *IncX1* plasmids carrying *bla, aad*, and *qnr* genes as well as heavy-metal resistance determinants, suggesting that diverse environmental pressures help maintain and disseminate these plasmids (Zalewska et al., 2024). Integrons, especially class I types, capture and express gene cassettes, promoting the accumulation and rapid reshuffling of resistance genes. Poultry isolates and manure samples frequently carry class I integrons, whose cassettes often include *aadA* and *dfrA*, reinforcing their role as major platforms for assembling resistance genes (Lemlem et al., 2024; Senthamilselvan et al., 2024). These integrons can circulate between cells and are commonly embedded in large conjugative plasmids, enhancing transmission across bacterial groups. Transposons and insertion sequences add genomic flexibility by mediating cut-and-paste or copy-and-paste movement of ARGs between chromosomes and plasmids. These rapid rearrangements can alter resistance-gene combinations and shift pathways of gene flow. In poultry production settings, ARGs often occur alongside elements such as *ISCR1* or *Tn3*-family transposons, which enable carbapenemase or *ESBL* genes to move onto new plasmids, broadening their host range and altering transmission patterns (Darphorn et al., 2021).

**Table 2:** Classification and characteristics of MGEs.

**MGEs class**	**Representative**	**ARGs types**	**Main mechanisms**	**Common hosts**	**Characteristics in poultry gut and environment**
Plasmids	*IncF, IncI1, IncX, IncC*	*blaCTX-M, mcr-1, tetA, sul1*	Mediate HGT both within and across bacterial genera through conjugation; serve as key vectors for multidrug resistance islands.	*Escherichia, Klebsiella, Salmonella*	Widely detected in broiler intestinal tracts and poultry-farm wastewater; frequently harbor multidrug resistance regions facilitating rapid ARGs dissemination (Lemlem et al., 2024; Zalewska et al., 2024).
Integrons	class I integron (*intI1, aadA, dfrA*)	*sul1, aadA, qacEΔ1*	Capture, rearrange, and express gene cassettes, enabling stepwise ARGs accumulation and promoting genetic “platform” formation for ARGs polymerization.	*Escherichia, Enterobacter, Proteus*	Function as core nodes of HGT networks; abundance strongly correlates with ARGs prevalence in poultry production environments (Kalantari et al., 2021).
Transposons	*Tn3, Tn21, Tn916*	*tetM, ermB, sul1*	Excise and reinsert between chromosomes and plasmids, promoting inter-replicon mobility of ARGs and facilitating co-selection under antibiotic exposure.	*Enterococcus, Clostridium, E. coli*	Enriched in broiler fecal samples under high antibiotic pressure; abundance patterns strongly reflect selective intensity in production systems (Zahoor et al., 2024).
Integrative and Conjugative Elements	*SXT/R391* family, *ICEBs1*	*blaOXA, tetX, ermB*	Combine site-specific integration with conjugation-mediated transfer; integrate stably into host genomes while maintaining mobility potential.	*Enterococcus, Streptococcus, E. coli*	Co-evolve with antimicrobial resistance genes, enhancing host stress tolerance, and environmental adaptability (Sun et al., 2022; Zheng et al., 2023).
Phage	prophage, λ-like elements	*blaCTX-M, mefA, tetW*	Transfer ARGs via transduction, enabling dissemination independent of cell-to-cell contact.	*Salmonella, E. coli, Streptococcus*	Frequently detected in poultry manure and surrounding environmental matrices; may act as environmental ARGs reservoirs (Anastassopoulou et al., 2025).
Insertion sequences	*IS26, ISCR1, IS1216*	*mcr-1, blaNDM, tetA*	Drive ARGs recombination, copy-number expansion, and formation of composite transposons; serve as powerful amplifiers of multidrug-resistant regions.	*E. coli, Klebsiella, Enterococcus*	Promote assembly of multidrug-resistance islands in poultry-associated microbiota; enriched in both gut and environmental samples (Tang et al., 2024; Wang and Dagan, 2024).

## 3 Antibiotic usage and its effects on the poultry gut microbiome and resistome

### 3.1 Patterns of antibiotic application in poultry production

Antibiotic use in poultry differs widely by region, production scale, and management style, and includes therapeutic treatments, prophylactic or metaphylactic dosing, and the historically common use of antibiotics as growth promoters. Tetracyclines, macrolides, fluoroquinolones, sulfonamides, aminoglycosides, and β-lactams are among the antibiotic classes most frequently used in poultry and form major components of the poultry resistome (Ma et al., 2023). Tetracycline-related ARGs frequently make up the largest proportion of cecal resistomes, a pattern consistent with long-standing selective pressure (Yang et al., 2022). Poultry *in vivo* studies consistently demonstrate that shifts in gut microbial community structure are accompanied by corresponding changes in ARG profiles, particularly under antibiotic interventions. How strongly the resistome is disrupted depends on the antibiotic class as well as the dose, route of administration, and production stage (Dongre et al., 2025). For instance, the fluoroquinolone enrofloxacin can cause nonlinear changes in microbiome composition and ARGs patterns, and some low-dose regimens disturb the community and expand ARGs diversity more than higher doses (Temmerman et al., 2022). These findings underscore that long-term subtherapeutic exposure can destabilize the microbiota, reduce important commensals, and open ecological niches for ARGs-carrying taxa. Longitudinal studies show clear differences between growth-promoter and therapeutic antibiotic use: although microbial communities often recover partly after withdrawal, ARGs levels decline much more slowly, suggesting ecological memory and reservoir effects (Gupta et al., 2021). Stage-specific antibiotic practices also influence how ARGs accumulate over time and across gut regions. Early-life exposure, when the microbiome and immune system are still forming, increases downstream resistome signals, while treatments given near finishing heighten the risk of transmission through the food chain (Williams et al., 2023). Limiting antibiotic classes, shortening treatment courses, avoiding prolonged subtherapeutic use, and incorporating resistome monitoring at key production stages to reduce ARGs accumulation and spread (Ma et al., 2023).

### 3.2 Alterations in gut microbial ecology under antibiotic pressure

**Figure 2:**
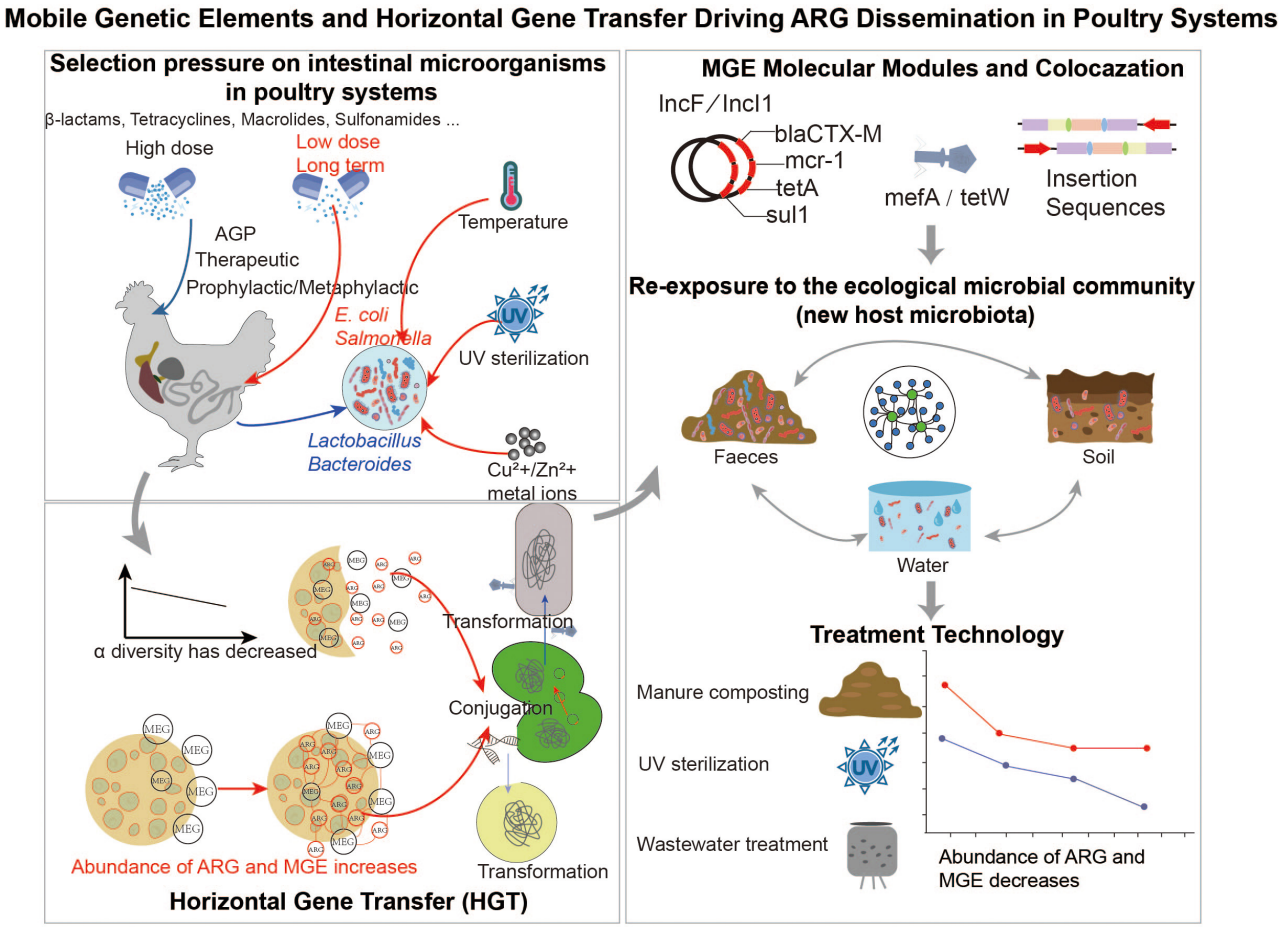
The figure summarizes the major environmental and management drivers underlying resistome expansion. Diverse antibiotic-use patterns exert selective pressure with non-linear dose–response relationships. Including the “low-dose paradox,” which amplifies ecological disturbance. Co-selective factors further enrich ARGs-carrying bacteria. Persistent ARGs enrichment reflects ecological memory. The schematic highlights a mechanistic chain linking antibiotic pressure, MGEs mobilization, ARGs proliferation, and environmental persistence.

Introducing antibiotics into the poultry gut triggers ecological disruptions that go far beyond selecting resistant strains, reshaping microbial composition, network structure, metabolic activity, and ARGs expression (Figure 2). Multi-omics studies indicate that even brief prophylactic exposure to fluoroquinolones or florfenicol can markedly expand the resistome. Therefore, the concept of “low-dose antibiotic paradox” was proposed. It refers to the observation that, when exposed to sub-therapeutic antibiotic concentrations over a long period of time, the damage to the intestinal microbial ecology and the enrichment of ARGs is the same or even greater. For example, studies in riverine biofilm communities exposed to sub-MIC antibiotics report upregulation of mutagenesis and horizontal gene transfer pathways, facilitating the spread and prevalence of ARGs under continuous low-dose selection pressures (Bourdin et al., 2025; Flores-Vargas et al., 2023). Early-life antibiotic treatment produced about 0.52 ARGs copies per cell across 13 classes and 72 subtypes, resulting in a noticeably enriched resistome in treated birds (Liang et al., 2023). Typical community responses include reduced α-diversity, shifts in β-diversity, loss of major commensals, and increases in tolerant or opportunistic taxa, especially Proteobacteria and MGEs-associated strains. Dose–response patterns are often nonlinear, prolonged low-dose exposure may disrupt microbial balance more strongly than short-term high-dose treatment, leading to higher ARGs diversity and abundance (Temmerman et al., 2022). In weaned piglets given low-dose antibiotics for 4 weeks, transcriptomic analyses revealed altered immune-related gene expression and changes in the abundance of specific transferable ARGs, implying potential impacts on resistance dissemination despite minimal overall resistome shifts (Hu et al., 2020). Importantly, low-dose effects are not universal and depend on antibiotic class, exposure duration, microbial community composition, and host developmental stage. In many cases, resistome perturbations appear transient, whereas in others they persist due to ecological memory and co-selection mechanisms. Antibiotics also alter the gut metabolic environment, and these shifts can influence both ARGs expression and their transfer potential. For example, enrofloxacin changes cecal SCFAs levels and host metabolic pathways, and these changes correspond with increases in fluoroquinolone-linked ARGs (Ma et al., 2023). In many cases, functional resistance responses, including increased expression of ARGs and efflux systems, appear before visible shifts in community structure, suggesting that metabolic and transcriptional signals may act as early markers of resistome expansion. Studies of antibiotic growth promoters show a similar pattern, ARGs and efflux gene expression increases even when community structure changes only slightly (Paul et al., 2022). Network analyses also show that antibiotic pressure disrupts microbial modularity and shifts previously low-abundance but mobile ARGs nodes into central positions within the network (Błażejewska et al., 2022). From an ecological and intervention standpoint, several key points stand out. First, surveillance systems should move beyond static catalogs of ARGs-carrying taxa and instead incorporate dynamic indicators such as transcriptional activity, metabolic alterations, and network centrality to enable earlier risk detection. Second, stewardship programs must consider both dosing and timing, avoiding long-term subtherapeutic use that can cause greater ecological disruption. Third, combining multi-omics approaches with network modeling is critical for understanding how resistomes are rebuilt and for designing targeted microecological interventions.

### 3.3 Persistence and co-selection of ARGs

In poultry systems, both gut and environmental resistomes often fail to return to pre-intervention levels after antibiotics are withdrawn. This persistence largely reflects two linked mechanisms: co-selection and ecological memory. Co-selection arises when stressors unrelated to antibiotics, such as heavy metals, disinfectants, or other farm chemicals, continue to select for bacteria carrying ARGs. Because ARGs often appear alongside metal-resistance genes on plasmids or integrons, strains capable of tolerating both antibiotics and metals can survive and spread even when antibiotics are no longer present (Bombaywala et al., 2021). Studies report strong correlations between ARGs patterns and levels of metals such as cadmium, arsenic, zinc, and copper. A multi-farm survey even found that metal concentrations influenced resistome composition more than antibiotic residues, underscoring the central role of metal-driven co-selection (Paul et al., 2022). Ecological memory describes stable community states that develop after long-term or repeated disturbances, including chronic low-dose antibiotic exposure. These altered states can keep ARGs levels high by locking in niche occupation patterns, stabilizing MGEs, and shifting host–microbe interactions. Long-term monitoring shows that certain ARGs and resistant strains persist even after antibiotic use declines, indicating that withdrawal alone is not enough to rapidly reduce resistome levels and that broader, system-level management strategies are needed (Smith et al., 2023). Manure-handling practices at the farm level also affect how long ARGs persist and how widely they spread in the environment. Standard composting or storage typically reduces ARGs only partially and may even concentrate them in remaining solids. In contrast, thermal treatment and optimized anaerobic digestion can greatly reduce ARGs and MGEs, although their effectiveness depends heavily on factors such as temperature, retention time, and substrate composition. The continued presence of high-risk ARGs after treatment underscores the need for standardized protocols and strict post-treatment verification (Flores-Orozco et al., 2024).

## 4 Transmission pathways of ARGs in poultry production systems

### 4.1 Vertical transmission

**Figure 3:**
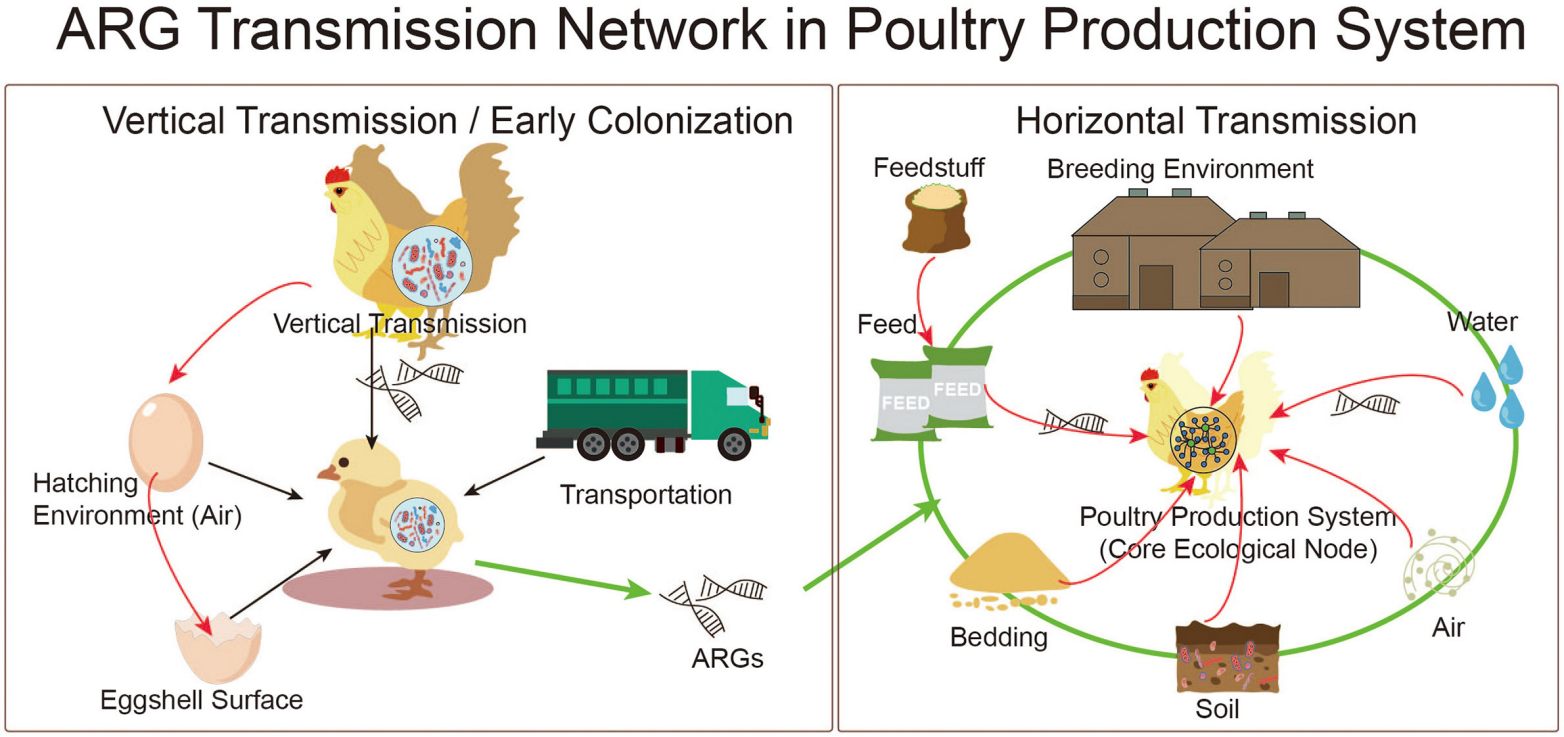
ARGs transmission network within poultry production systems. The model illustrates multiscale ARGs transmission routes. Vertical transmission occurs via maternal transfer, hatchery contamination, and early-life colonization. Horizontal transmission occurs via feed, drinking water, litter, air, and biofilms. Environmental dissemination via poultry manure contributes to soil, water, and crop contamination. These pathways form feedback loops that reintroduce ARGs into animals and humans. Network analysis highlights MGEs-mediated gene exchange between commensal and pathogenic bacteria.

The brooding period represents a key stage during which the gut microbiota forms and the initial resistome becomes established (Ahmad et al., 2023). Microbes in the hen's reproductive tract influence both internal and external egg microbiota and can pass ARB or MGEs to embryos, shaping the baseline resistance profile of newly hatched chicks (Wang and Chai, 2022; Figure 3). When the reproductive tract or ovaries of hens are colonized by resistant strains, ARGs can be deposited in the egg before laying, enabling true vertical transmission and resulting in chicks hatching with previously established specific resistant lineages or gene clusters (Fan et al., 2025). Day-old chicks often serve as asymptomatic carriers of ARB. For instance, a survey of 180 Belgian chicks found *E. coli* and *Enterococcus* isolates containing *sulI, sulII*, and *bla_TEM*, showing that chicks can introduce resistance into production systems even without symptoms (Dougnon et al., 2023). Metagenomic source tracking showed that by day 5, roughly 57% of cecal ARGs came from feather microbiota and about 30% from the cage environment, highlighting how strongly hatchery and brooding environments contribute to early resistome development (Xiao et al., 2023). ARGs acquired early in life may later spread horizontally via plasmids, integrons, or other MGEs within the gut, increasing the overall resistance burden in the flock. These resistant strains can spread through feces, litter, and dust, strengthening environmental reservoirs and raising downstream public health risks. Therefore, breeding and hatchery practices represent upstream control points that can either amplify or reduce AMR risks along the production chain (Abreu et al., 2023). To reduce early colonization by unwanted strains, microbiota-based approaches, such as probiotics or targeted microbial transplantation, can help beneficial taxa establish early and limit pathogen or ARGs establishment (Zhao et al., 2025). Such strategies support gut homeostasis and lower pathogen loads during early development. Likewise, qPCR- or metagenomics-based monitoring of ARGs and MGEs at the breeding hatching brooding interface can help assess risks and evaluate intervention effectiveness (Wang and Chai, 2022).

### 4.2 Horizontal transmission: within and between flocks

In poultry systems, horizontal transmission is the main route by which ARGs persist and spread (Figure 3). Biofilms in drinking-water lines also form persistent ARGs reservoirs, *intI1* and *bla_CTX-M* have been detected in more than 60% of poultry water systems (Chen Y. et al., 2025; Mak et al., 2022). Litter and feces are high-risk materials in which ARGs accumulate, change, and recirculate. Metagenomic studies have identified more than 180 ARGs types in litter, mainly *tet, sul*, and *erm* genes, and their abundances strongly correlate with MGEs such as *tnpA* and *intI1*. Reusing litter intensifies this burden, with *tetW* and *sul2* levels rising as much as 3.5-fold compared with fresh bedding (Farooq et al., 2022). Airborne transmission represents another pathway, often underestimated. Dust and aerosols in poultry houses can carry ARB and ARGs over considerable distances (Xin et al., 2023). Around 70% of monitored ARGs can be detected in indoor aerosols, and their levels are affected by stocking density, litter properties, and ventilation. Within the gut microbiota, gene exchange further supports ARGs maintenance (Chen Y. et al., 2025; Xu X. et al., 2022). Commensals like *Enterococcus* and *Lactobacillus* commonly exchange plasmids with pathogens such as *E. coli* and *Salmonella*. Transfer of *bla_TEM-1* from *E. coli* to *Salmonella* can reach frequencies of 10^−3^ and is often accompanied by increased integron copy numbers (Scicchitano et al., 2024). These findings show that ARGs circulation can continue within microbial communities even when external antibiotic pressure decreases. Horizontal transmission forms a connected network across feed, water, litter, air, and gut microbial interactions (Ding et al., 2019; Koorakula et al., 2022; Paul et al., 2022). Mitigating this network requires a systems-based approach, improving feed and water hygiene, optimizing litter treatment and replacement, managing air quality, and limiting gene exchange within the gut to disrupt transmission routes and lower production and public health risks.

### 4.3 Environmental dissemination

Poultry manure and farm wastewater are major routes through which ARGs enter the environment. When inadequately treated manure is applied directly to cropland or stored on-site, it markedly increases the abundance and diversity of soil ARGs, turning soils into long-term resistant reservoirs. These ARGs may enter the food chain through plant uptake or surface contamination, creating re-exposure risks for animals and humans (Wang et al., 2023). Manure brings large amounts of ARGs, ARB, and co-selective agents such as antibiotics and heavy metals, while soil microbes serve as recipients that can acquire and propagate these genes through plasmids, integrons, and transposons. The abundance of *tet* and *sul* resistance genes increases sharply after the application of manure and may persist for months to years, with untreated manure generating substantially higher residual ARGs levels than composted or anaerobically digested material (Zhang et al., 2024). Farm wastewater represents another major route for spreading ARGs across water bodies. Wastewater containing bacteria, antibiotic residues, and MGEs can spread ARGs to surface water, groundwater, or wetlands when discharged without adequate treatment. Monitoring frequently detects *sul1, tetM*, and *blaCTX-M* at livestock effluent points and downstream locations, and their abundance often correlates with production intensity. Even after treatment, wastewater effluents can still contain detectable ARGs, contributing to ecological risks downstream (Li et al., 2025). From a One Health perspective, wastewater forms a key link between animal production, environmental contamination, and human exposure (Franklin et al., 2024). Environmental ARGs also show persistence and feedback potential. Soil, water, and crops form connected pathways that allow ARGs to circulate across environments. Birds, wildlife, and farm workers can also act as carriers, moving ARGs across ecological boundaries and increasing cross-system health risks (Ahmad et al., 2023).

## 5 Sustainable control and mitigation strategies

### 5.1 Microbe-based approaches

**Figure 4:**
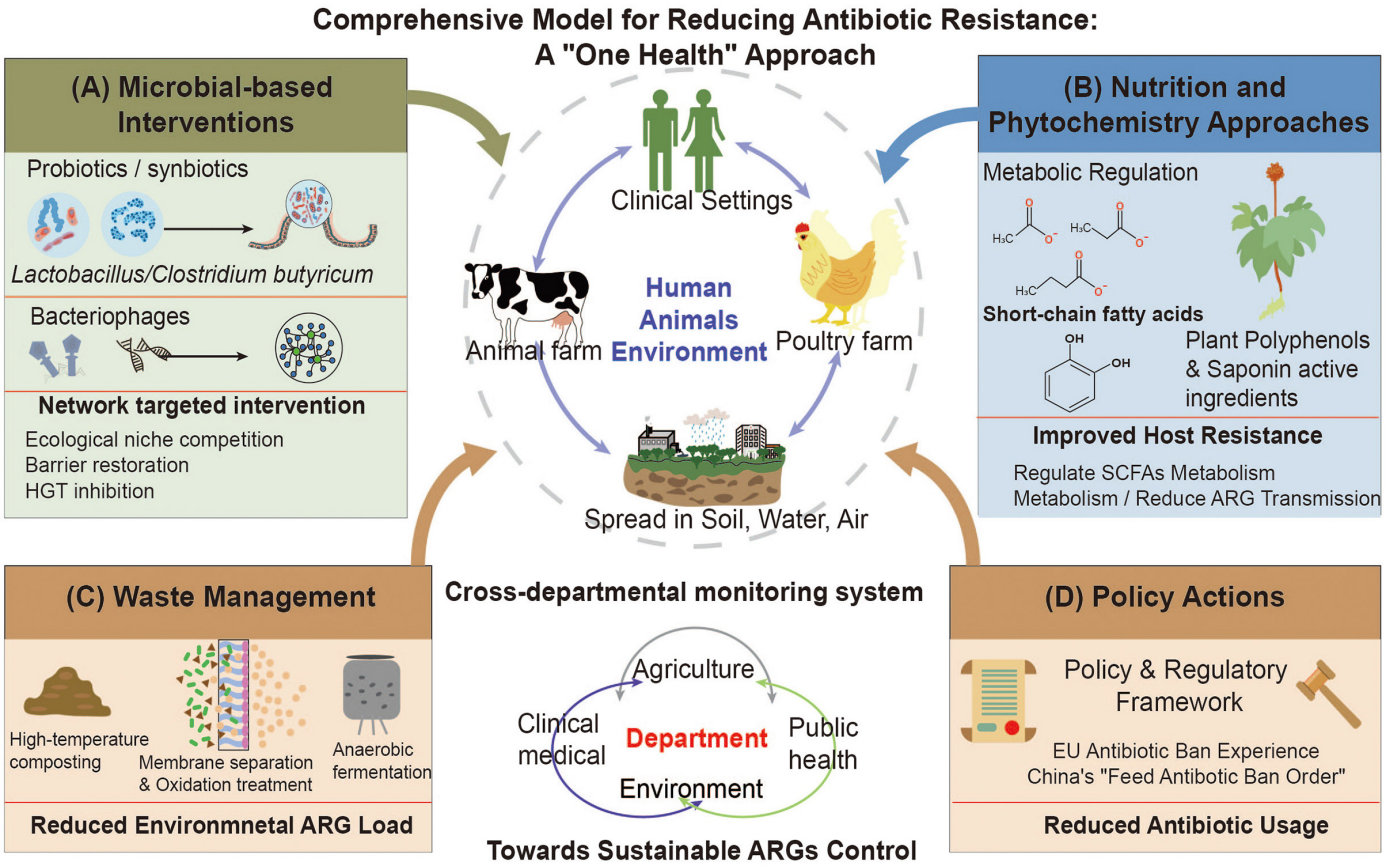
An integrated mitigation model is proposed to reduce the poultry gut resistome. The framework brings together three synergistic strategies. **(A)** Microbe-based interventions, including probiotics, synbiotics, and phage cocktails, enhance ecological competition, improve barrier integrity, and limit HGT. **(B)** Nutritional and phytochemical approaches, such as SCFAs, polyphenols, and saponins, are highlighted for their ability to modulate metabolic pathways, regulate SCFAs metabolism, and mitigate ARGs dissemination. **(C, D)** Waste management and policy actions, including thermophilic composting, anaerobic digestion, and national AMR policies (EU and China), are shown to reduce environmental ARGs reservoirs. Overall, the model supports a mitigation framework aligned with the One Health concept.

Microbiota-based interventions can reduce ARGs abundance and transmission by altering the structure and activity of gut and environmental microbial communities. These strategies offer targeted and long-lasting mitigation options compatible with poultry production, including competitive exclusion via probiotics or synbiotics, phage-based removal of specific pathogens, and multi-omics–guided network regulation (Figure 4A). Probiotics and synbiotics limit ARGs spread through niche competition, improved barrier function, and metabolic adjustments. When probiotics successfully colonize the gut, they take up key spatial and nutritional niches, discouraging colonization by pathogens or ARGs-bearing bacteria. Supplementation with *Lactobacillus* or *Bacillus* species can reshape the gut community and reduce the abundance of major ARGs carriers (Chen Y. et al., 2025). Probiotics can also enhance epithelial integrity by increasing tight junction proteins, mucins, and immune markers, which lowers gut permeability and limits opportunities for plasmid- or integron-mediated HGT. Studies in chickens show that *Clostridium butyricum* or lactic acid bacteria can improve barrier indicators and reduce *tet* and *sul* gene abundance (Liu et al., 2023). Synbiotics combine probiotics with prebiotics such as oligosaccharides or cellulose, improving colonization stability and boosting SCFAs production. SCFAs lower gut pH and suppress ARB growth while downregulating genes involved in HGT, producing combined ecological and metabolic benefits. Synbiotics often lead to more stable reductions in gut ARGs levels; for example, adding *Lactobacillus plantarum* to feed or silage significantly reduced both absolute and relative levels of several ARGs (Chen Y. et al., 2025). Bacteriophages, with high host specificity and strong lytic activity, can selectively eliminate resistant pathogens such as *ESBL*- or *bla_CTX-M*–positive *E. coli*. Trials show that both single-strain and cocktail phage preparations can substantially reduce target bacteria *in vitro* and *in vivo* (Koratkar et al., 2024). However, phage use has limitations, such as poor stability, the emergence of phage-resistant mutants, and the risk of phage-mediated ARGs transfer through specialized or generalized transduction (Abd-El Wahab et al., 2023). Thus, phage therapy requires genome-level checks, careful evaluation of transduction potential, and assessment of long-term ecological risks to avoid unintentionally promoting ARGs spread (Chen W. et al., 2025; Pilati et al., 2023). Multi-omics–guided network approaches use microbial interaction maps to identify influential taxa and critical MGEs that strongly shape ARGs flow. Targeted probiotics or phage removal of these taxa has disrupted ARGs transmission modules in small-scale studies, marking progress toward precision resistome management (Kumar et al., 2025). However, probiotic strains may themselves harbor intrinsic or acquired ARGs, raising concerns about inadvertent gene dissemination. For example, next-generation sequencing of commercial poultry probiotic products has revealed that certain probiotic strains carry diverse antimicrobial resistance genes, including determinants associated with fluoroquinolone, macrolide, and aminoglycoside resistance even when not originally selected for resistance traits, raising concerns about inadvertent ARG dissemination via probiotic supplementation (Kerek et al., 2024). During the use of probiotics and phage, the potential risk of probiotics carrying resistance genes needs to be evaluated (Soffritti et al., 2025). Further conducting research on the molecular genetics of probiotics is of great significance for reducing the risk of resistance gene transfer in agriculture and ensuring the safe and effective application of probiotics in the livestock industry.

### 5.2 Nutritional and phytochemical interventions

**Figure 5:**
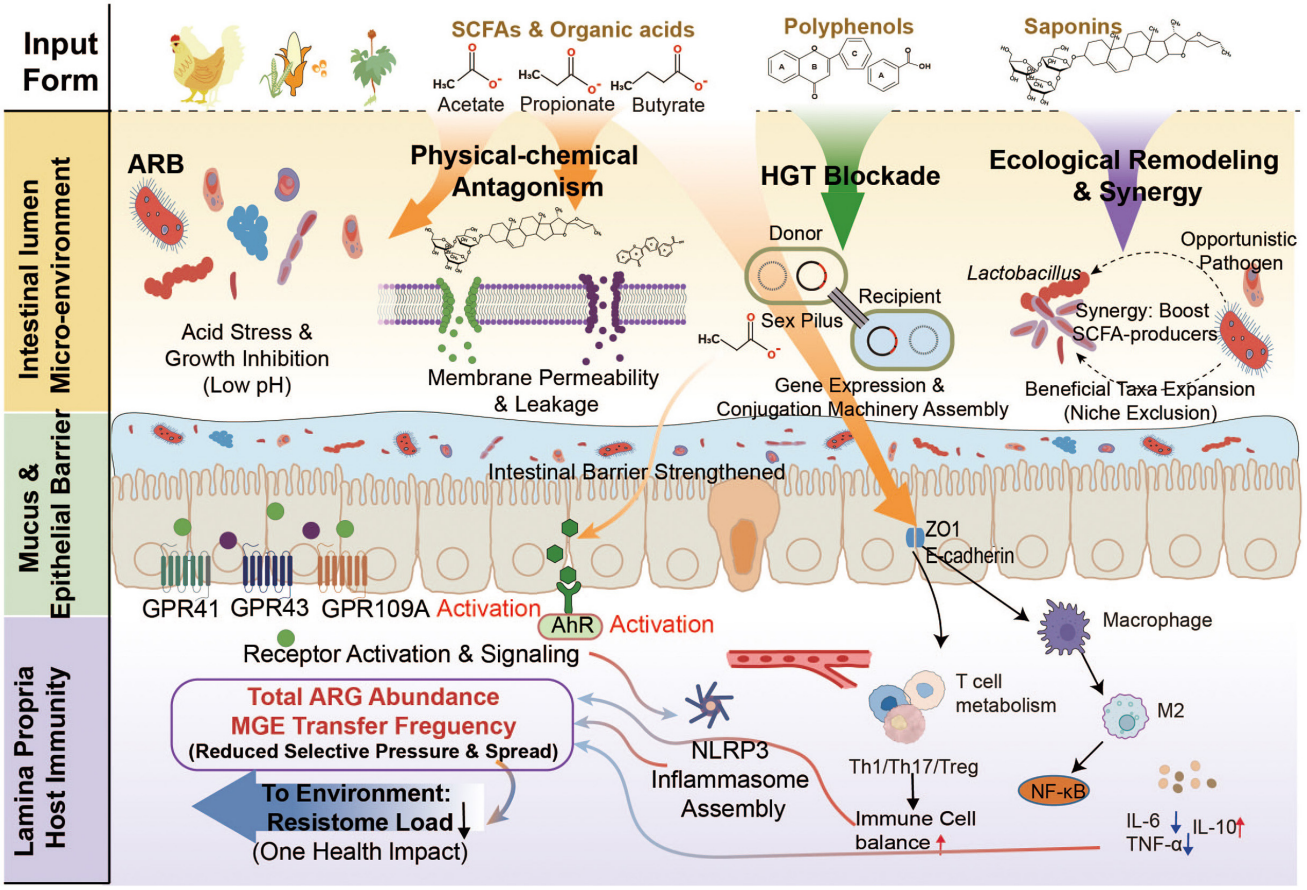
The diagram depicts the multitarget actions of SCFAs, polyphenols, saponins, and organic acids. These compounds generate an acidic microenvironment and disrupt bacterial membranes, directly suppressing ARB proliferation. Metabolites such as butyrate downregulate conjugation machinery, thereby limiting plasmid-mediated HGT. These interventions favor beneficial taxa that competitively exclude opportunistic ARGs carriers. SCFAs upregulate ZO-1 and Occludin, strengthening epithelial integrity. At the molecular level, these phytochemicals activate GPR43 and AhR receptors while modulating NF-κB and NLRP3 inflammatory pathways. This immunomodulatory effect reduces inflammatory niches that favor resistant pathogen expansion.

Nutritional and phytochemical interventions offer environmentally sustainable options for limiting ARGs proliferation by modulating the gut microenvironment and host–microbe interactions (Figures 4B, 5). Instead of acting exclusively on bacterial populations, these compounds influence lumen acidity, metabolic outputs, epithelial integrity, and immune signaling at a system level, thereby constraining ARGs persistence and HGT. Plant-derived bioactives regulate host inflammatory pathways, especially those involving AhR and NF-κB, thereby stabilizing mucosal immunity and reducing ecological niches available to resistant strains (Feng et al., 2022; Soffritti et al., 2025). Likewise, diet-induced changes in microbial fermentation enhance SCFAs availability, which can strengthen epithelial barrier integrity and limit contact dependent gene exchange (Li et al., 2023). Evidence from poultry models indicates that nutritional modulation reduces ARGs abundance while promoting more resilient, SCFAs-enriched microbial communities (Tan et al., 2025). For example, an *in vitro* bacterial conjugation assay demonstrated that the presence of butyrate derivatives reduced conjugation frequencies between different donor–recipient pairs by approximately 3–8 fold. This reduction was associated with decreased expression of outer membrane proteins and conjugation-related genes (*trfAp/trbBp*), potentially mediated through alterations in membrane permeability and activation of the CpxAR signaling pathway (Tan et al., 2025). In an *ex vivo* chicken intestinal explant model, exposure to SCFAs (acetate, propionate, and butyrate) at a concentration of 0.01 mol/L resulted in a significant reduction in plasmid-mediated resistance transfer, whereas near-complete inhibition was observed at higher concentrations ranging from 0.1 to 1 mol/L (Ott and Mellata, 2024). *In vivo* evidence further indicates that dietary fiber–induced increases in intestinal SCFAs levels can reduce the overall frequency of ARGs horizontal transfer by up to approximately 5.8 fold, accompanied by shifts in microbial community structure, including a decreased proportion of ARGs carriers within Firmicutes and Proteobacteria (Tan et al., 2025). However, such inhibitory effects are not consistently observed across studies. Accumulating evidence suggests that both the direction and magnitude of SCFAs-associated responses are highly dependent on microbial taxa, plasmid backbones, and environmental variables, including pH, carbon availability, and antibiotic selective pressure. For instance, distinct SCFAs exert concentration- and pH-dependent effects on the growth of *E. coli* and the expression of resistance-associated genes, with certain genes being upregulated at lower concentrations but suppressed only at higher levels, underscoring strong interactions between microbial metabolites and surrounding environmental conditions (Kadry et al., 2023). Therefore, although SCFAs exhibit the potential to constrain ARG conjugative transfer or modulate resistance gene abundance in selected *in vitro* and *in vivo* models, these effects are not universal and are strongly modulated by ecological and experimental contexts. Based on current evidence, SCFAs are more appropriately regarded as conditional ecological modifiers rather than deterministic inhibitors of antimicrobial resistance dissemination.

Importantly, changes in ARGs abundance, whether influenced by SCFAs or other ecological drivers, should be interpreted separately from actual AMR risk. A high ARGs load in microbiomes or environmental samples does not necessarily correspond to increased clinical resistance risk. This discrepancy arises because ARGs vary in mobility, host range, and pathogenic relevance, and only a small subset associated with mobile elements and pathogenic hosts poses meaningful public health concerns. Accordingly, recent risk frameworks integrate indicators such as human-associated enrichment, mobility through mobile genetic elements, and occurrence in pathogenic hosts, revealing that only a minority of ARGs families qualify as current high-risk genes (Qian et al., 2022; Zhang et al., 2022). These models highlight that ARGs abundance alone is an insufficient proxy for risk, particularly in complex systems where non-pathogenic hosts or inactive genes can inflate resistome estimates (Zhang et al., 2021). From a One Health perspective, ARGs surveillance and mitigation should prioritize genes with high transfer potential and clinical relevance, rather than relying solely on abundance-based metrics.

### 5.3 Ecological cycle and the management of manure

Manure and wastewater serve as major conduits for ARGs release into the environment, making their safe treatment and resource recovery essential for limiting the dissemination of ARGs (Figure 4C). As poultry manure has a high moisture content and fine particle size, it contributes to more rapid short-term accumulation of the *tet* and *sul* genes in soils, and these ARGs may persist for several months or longer if management is inadequate (Mecik et al., 2023). High temperature composting is an effective strategy for reducing ARGs in manure. Under controlled thermophilic conditions (>55–65°C for several days to weeks), the abundance of mobile ARGs typically decreases by 40–80%. Composting poultry manure significantly downregulates *tet, sul*, and *erm* gene expression and reduces overall mobile ARGs levels (Zhang et al., 2024). However, removal efficiency varies among different ARGs and MGEs types (e.g., *intI1*), highlighting the need to tailor composting parameters to specific gene risk profiles. Inadequate management during the late composting phase can lead to ARGs rebound or enrichment of ARB, underscoring the importance of strict process control (Basil et al., 2024). Anaerobic digestion achieves partial ARGs reduction while simultaneously producing renewable biogas. Its effectiveness is strongly influenced by temperature, hydraulic retention time, and feedstock composition. Coupling anaerobic digestion with advanced treatments, including membrane filtration, advanced oxidation, or hybrid co-chemical processes, can substantially reduce ARGs and residual antibiotics in effluents and digestates (Basil et al., 2024). In large-scale wastewater treatment systems, biofilm reactors and sequencing batch reactors enhance microbial stability and pollutant removal. In contrast, membrane filtration and ozone disinfection further enhance the removal of high-risk ARGs and MGEs. In summary, properly treated poultry manure supports nutrient recycling in agricultural systems. However, strict processing standards, rational land-application guidelines, and long-term soil and crop monitoring are essential to prevent ARGs accumulation and downstream transmission through agroecosystems and the food chain (Chen Y. et al., 2025).

### 5.4 Management and policy-level actions

Limiting the dissemination of ARGs in poultry production requires a combination of farm-level technological measures and integrated management and policy frameworks. Core components include restricting antibiotic growth promoters, establishing cross-sector surveillance systems, and integrating manure valorization into broader environmental remediation strategies (Figure 4D). The 2006 ban on antimicrobial growth promoters in the European Union enabled long-term tracking of AMR indicators across animal, food, and human sectors. Recent EU surveillance reports indicate declining multidrug resistance in selected pathogens, accompanied by reduced sales of medically important veterinary antimicrobials [European Food Safety Authority (EFSA); European Centre for Disease Prevention and Control (ECDC), 2024]. In the United States, the National Action Plan for Combating Antimicrobial Resistance (2020–2025) integrates public health, agricultural, and environmental sectors under a One Health framework (Office of the Assistant Secretary for Planning and Evaluation, 2020). Since 2020, the ban on antimicrobial growth promoters in China has been accompanied by comprehensive regulatory frameworks covering veterinary drugs, feed additives, and environmental management. Complementary measures, including restrictions on veterinary antibiotic sales, the promotion of alternatives, and improvements in husbandry practices, are widely regarded as key drivers of AMR reduction in domestic livestock and poultry. Early surveillance data indicate improvements in selected regions and metrics following policy implementation. However, outcomes vary depending on enforcement intensity, access to alternatives, and local compliance. Sustained monitoring and transparent data sharing remain essential for evaluating long-term effectiveness.

## 6 Conclusion

The poultry gut resistome does not arise from a single driver, but instead represents an ecological feature shaped over time by interactions among microbial communities, mobile genetic elements, and the local metabolic and physiological environment. As a result, the persistence and spread of antimicrobial resistance cannot be explained by selective pressure alone, but also reflect microbial adaptation, gene mobility, and niche organization. Building on this view, we propose the RMM axis as a framework that views antimicrobial resistance as a dynamic process governed by intertwined metabolic and ecological forces. Several proposed interactions within the RMM axis are based on ecological theory and systems-level inference, integrating observed correlations rather than direct causal evidence. Within the RMM framework, interventions such as probiotics, prebiotics, and plant-derived bioactives can reshape microbial metabolites, particularly SCFAs, thereby influencing resistance gene expression, horizontal gene transfer, and microbial competition. Importantly, the RMM axis should not be interpreted as a fixed or deterministic pathway but as a flexible framework that integrates evidence of varying strength to guide future experimental validation in poultry systems. This perspective helps explain why resistance genes often persist after antibiotic withdrawal and how co-selection and ecological memory stabilize resistance across gut and environmental contexts. Accordingly, future resistance risk assessments should prioritize functional indicators, including metabolic states and transcriptional activity, to allow earlier detection of resistome expansion. From a One Health perspective, effective mitigation requires integrating gut ecological processes with manure management and environmental feedbacks. By redirecting attention from individual resistance genes to the ecological processes that sustain them, the RMM axis offers a foundation for more durable resistance control strategies in poultry systems.

## 7 Challenges and future perspectives

Future AMR research in poultry needs to move beyond single-layer profiling and adopt a systems-level RMM axis framework that can better pinpoint the real drivers of ARGs amplification and identify feasible intervention targets. Coordinated analysis of metagenomic, metabolomic, and transcriptomic or metatranscriptomic data can clarify the phylogenetic sources of ARGs, their regulatory behavior, and their ecological roles (Hu et al., 2024). When combined with causal modeling and perturbation experiments this approach helps reveal how metabolic activities influence ARGs activation, MGEs mobility, and resistance spread. Another priority is linking precision nutrition with targeted modulation of the microbiome. Rather than focusing only on community composition, precision biotics and other function-oriented strategies seek to reprogram metabolic pathways and signaling networks that influence ARGs amplification. Building causal ARGs metabolite microbe interaction networks, supported by machine learning and multi-omics graph models, will be key to identifying influential metabolites, high-risk microbial hosts, and MGEs that strongly shape resistome trajectories (Yan et al., 2023). At the applied level, green alternatives require systematic assessment of dose-response patterns, functional effectiveness, safety, and cost-benefit performance (Rahman et al., 2022). Evaluating these interventions within the RMM axis framework will make it possible to assess their long-term impacts on resistance network structure, ARGs expression dynamics, and cross-host transmission. Resistance governance also needs to be integrated into a One Health framework, backed by cross-sector surveillance and open data sharing. Global AMR platforms should expand their coverage to include livestock, manure, and environmental samples, allowing interoperable human–animal–environment datasets to support cross-border source tracking, risk assessment, and intervention optimization. Interpretation of poultry resistome data is strongly influenced by sampling strategy and matrix selection. Cecal samples typically display greater microbial diversity and higher ARGs richness than fecal samples, which mainly reflect distal gut contents and transient microbial shedding. Environmental matrices such as litter, dust, and water constitute distinct ecological niches shaped by external selective pressures and often harbor resistome profiles that differ from those within the gastrointestinal tract. As a result, resistome patterns obtained from different sample types are not directly comparable, and cross-study comparisons should be interpreted cautiously. Bringing ecological network analysis, metabolic function profiling, and ARGs risk prediction together into a unified platform could offer the poultry industry an early-warning-capable governance system. The RMM axis emphasizes the interconnected roles of genes, microbes, and metabolites in shaping ARGs emergence and spread, providing a strong basis for precision control and integrated One Health management.

## 8 Review methodology

This review was conducted as a structured narrative synthesis of the literature focusing on antimicrobial resistance in poultry production systems. Relevant studies were identified through searches of Web of Science, PubMed, and Scopus, covering publications from approximately 2005–2025. Search terms included combinations of poultry, chicken, gut microbiota, resistome, antibiotic resistance genes, mobile genetic elements, horizontal gene transfer, and microbial metabolites. Priority was given to poultry-specific *in vivo* and *in vitro* studies, particularly those examining ARG dynamics, MGEs, and host–microbiome interactions under production-relevant conditions. Non-poultry or environmental studies were included when they provided mechanistic insights relevant to RMM interactions and were explicitly identified as extrapolative. Studies were selected based on relevance, methodological rigor, and contribution to understanding resistance transmission and risk rather than exhaustive coverage of all reported ARGs or taxa.
